# Solitary splenic metastasis of ovarian carcinoma: a case report

**DOI:** 10.1186/1752-1947-8-154

**Published:** 2014-05-17

**Authors:** Dror Karni, Doron Kopelman, Ossama Abu Hatoum

**Affiliations:** 1 Department of Surgery, Haemek Medical Center, Yitshak Rabin Boulevard, 18101 Afula, Israel; 2 Faculty of Medicine, Technion-Israel Institute of Technology, Technion City, Haifa 3200003, Israel

**Keywords:** Spleen, Ovary, Carcinoma, Metastasis

## Abstract

**Introduction:**

Splenic metastasis from ovarian carcinoma generally presents as peritoneal spread with multiorgan involvement. Fewer than 30 cases of solitary parenchymal splenic metastasis from ovarian carcinoma have been published in the literature. The presentation is often asymptomatic.

**Case presentation:**

An increase in tumor marker CA-125 from 18.1 to 132.6 units/ml (normal <35 units/ml) was measured in a 56-year-old Israeli Jewish woman who had undergone, six years previously, a total abdominal hysterectomy with bilateral salpingo-oophorectomy due to right ovarian carcinoma. An abdominal computed tomography scan revealed a mass of 6×8cm at the anterior of the spleen, with close proximity to the wall of the stomach. A gastroscopy demonstrated exterior pressure on the stomach body. An open splenectomy was performed to exclude a peritoneal carcinomatosis. No intraoperative evidence of tumoral spreading in the abdominal cavity was observed, other than the spleen. The final histologic result demonstrated a high-grade carcinoma consistent with metastatic endometrioid-type ovarian carcinoma grade 3.

**Conclusions:**

This case highlights the importance of cancer antigen 125 assessment and medical imaging in the follow-up of ovarian carcinoma. Open laparotomy, or laparoscopy, enables exclusion of a peritoneal carcinomatosis, which is more common than solitary parenchymal splenic metastasis, as was presented in the current case.

## Introduction

Metastasis of malignant tumors to the spleen is rare. Until recently the literature was based on autopsy studies, which reported frequencies of 2.3 to 7.1 percent [1,2] or splenectomy specimen studies, which reported a frequency of 1.3 percent [3]. According to data based on clinical course and evaluation, approximately 1 percent of malignant tumors metastasize to the spleen [4]. Splenic metastasis is generally part of multivisceral metastatic disease, with breast, lung, ovarian, colorectal and gastric carcinomas and skin melanoma the most common origins [5,6]. In Compérat *et al.*’s review of 93 cases published through 2006 [6], colorectal and ovarian carcinoma demonstrated the highest frequency as the primary source. Metastasis from ovarian carcinoma to the spleen is generally a part of peritoneal spreading with multiorgan involvement, generally involving the splenic capsule. Fewer than 30 cases of solitary parenchymal splenic metastasis from ovarian carcinoma have been published in the literature.

## Case presentation

A 56-year-old Israeli Jewish woman was admitted to our surgical ward for a splenectomy due to a solitary splenic mass. Six years prior to her admission she had undergone a total abdominal hysterectomy with bilateral salpingo-oophorectomy due to carcinoma in the right ovary. The histology examination revealed an endometrioid carcinoma, grade 3. Staging with International Federation of Gynecology and Obstetrics (FIGO) classification was 1A. Following the surgery, our patient received chemotherapy treatment with paclitaxel (Taxol®) and carboplatin.

**Figure 1:**
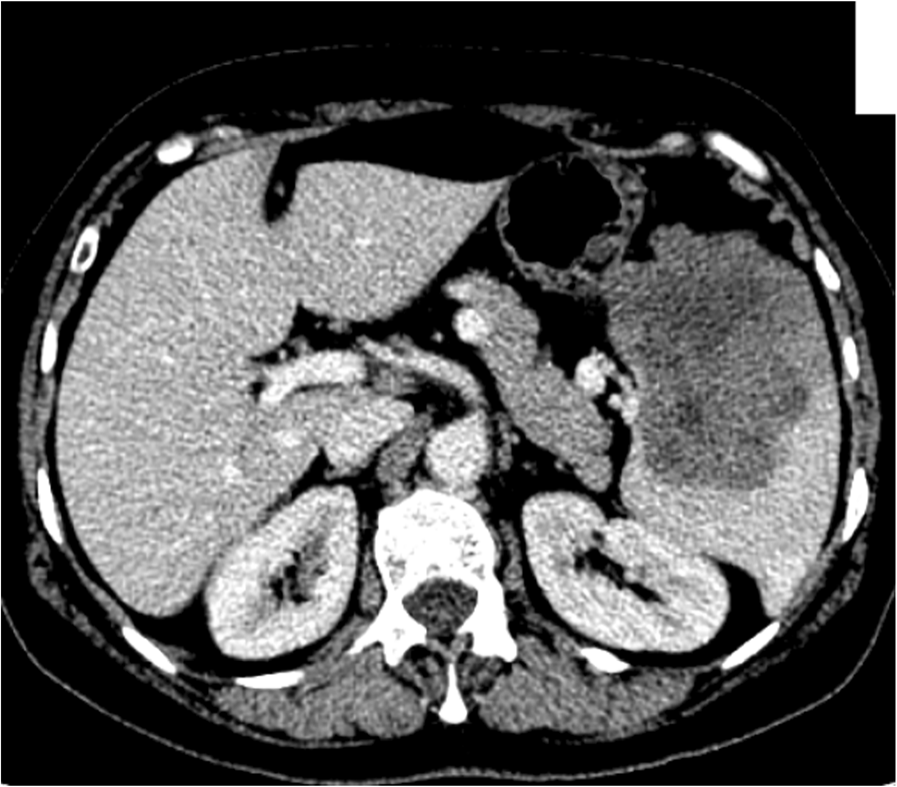
**Abdominal computed tomography.** Abdominal computed tomography revealed a heterogeneous hypodense mass of the spleen with suspected necrotic changes.

Tumor marker cancer antigen (CA)-125 was evaluated every year following the operation. Subsequent to a rise in CA-125 from 18.1 to 132.6 units/ml (normal <35 units/ml), our patient was referred for an imaging and an endoscopic assessment.An abdominal computed tomography (CT) scan revealed a mass of 6×8cm at the anterior of the spleen (Figure 1), with close proximity to the wall of the stomach. There was no other indication of a secondary tumor spreading in the abdomen. Her chest CT scan was normal. A gastroscopy demonstrated exterior pressure on the stomach body. All the specimens from gastric mucosa that were taken with the gastroscope and that were subjected to histologic testing were normal except a chronic inflammation in the gastroesophageal area. A colonoscopy also showed normal results. A bone isotopic scan revealed no abnormalities.

**Figure 2:**
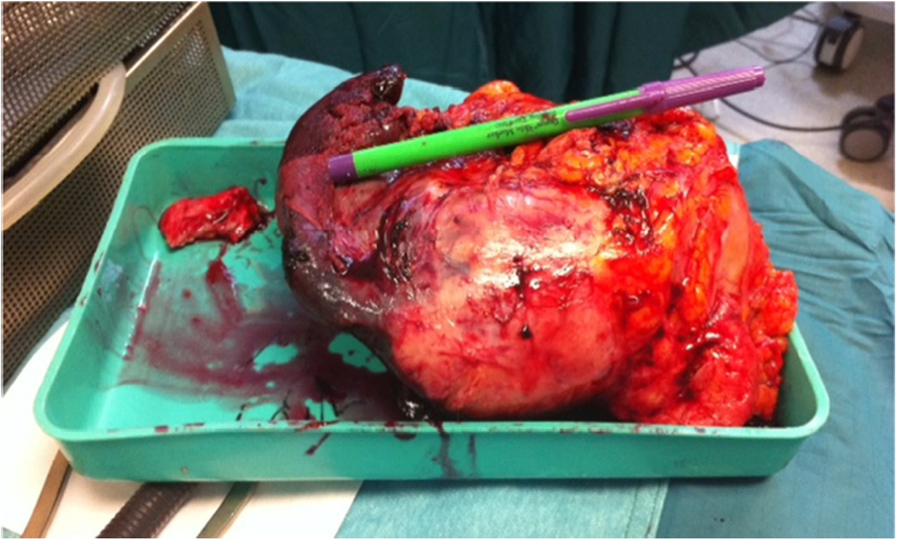
Spleen and adherent fat tissue specimen after resection.

**Figure 3:**
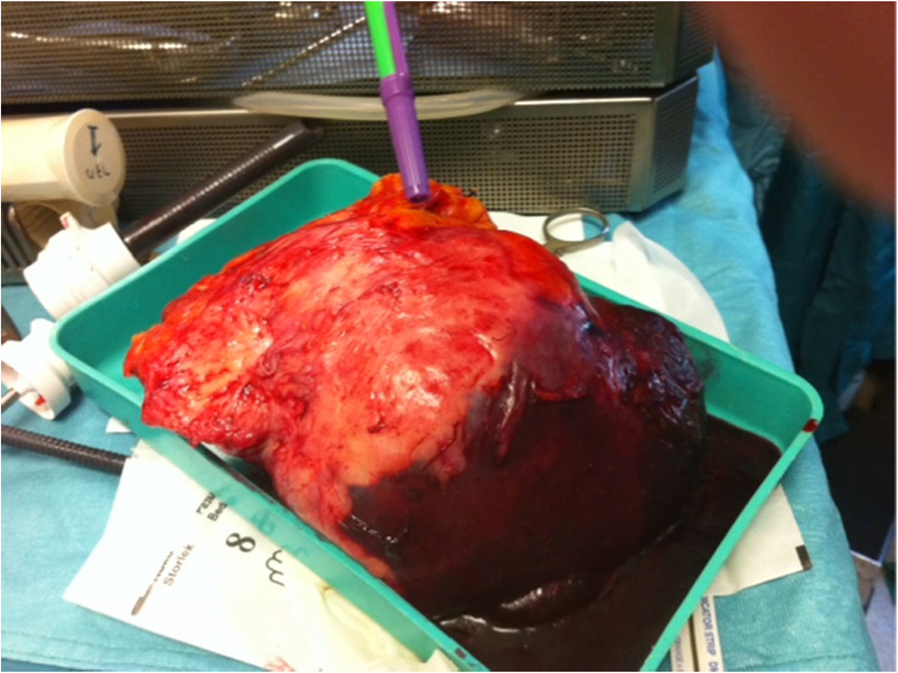
Spleen and adherent fat tissue specimen after resection.

**Figure 4:**
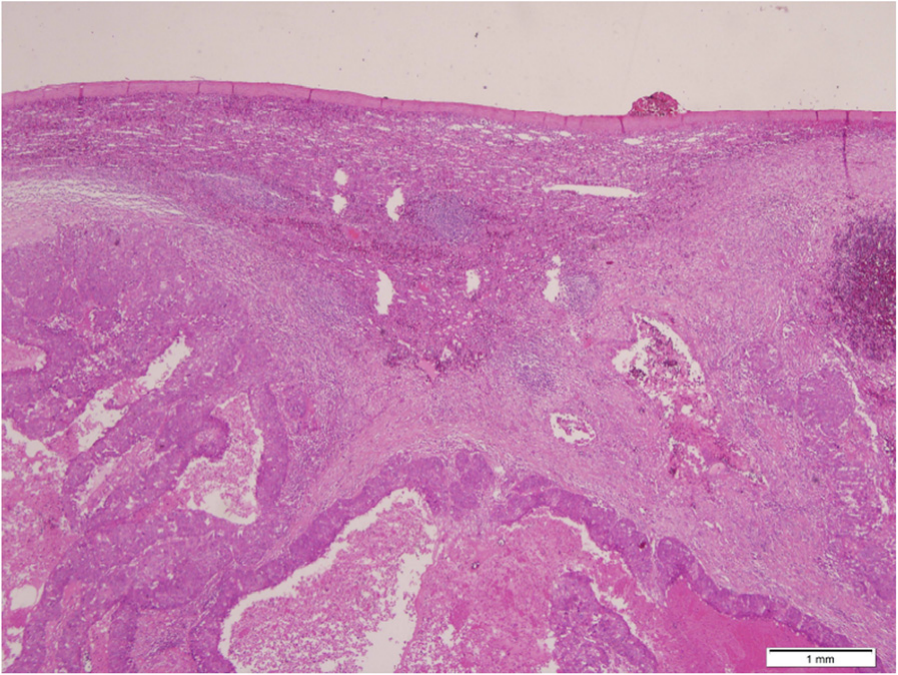
Splenic parenchyma with high-grade metastatic carcinoma (hematoxylin and eosin, ×40 original magnification).

**Figure 5:**
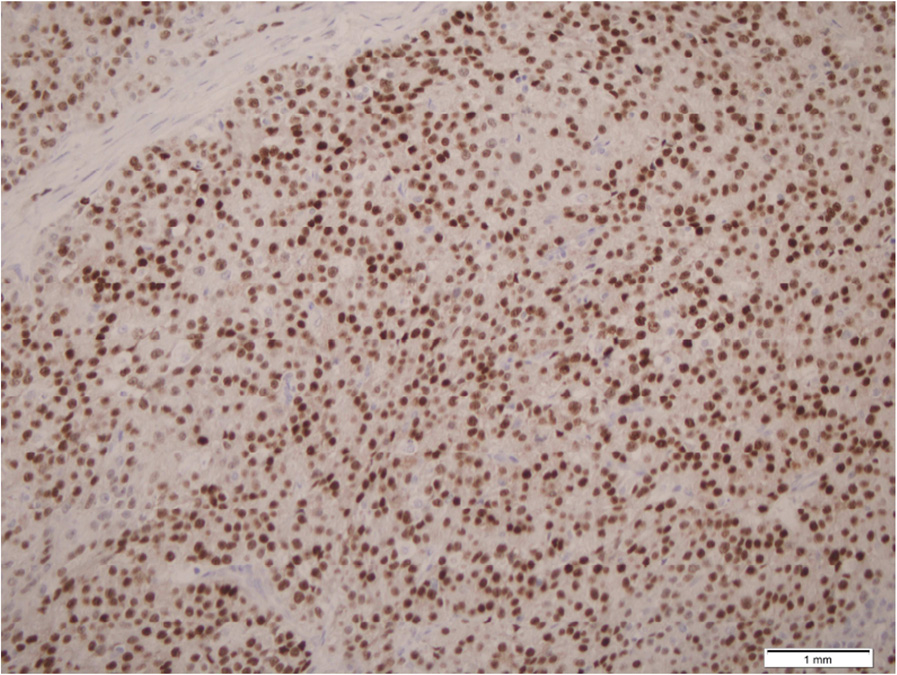
Metastatic ovarian carcinoma demonstrates strong positive estrogen receptor staining (estrogen receptor, ×200 original magnification).

Our patient was asymptomatic and her gynecologic and abdominal examinations were without any remarkable findings such as tenderness or splenomegaly.An open laparotomy through a left subcostal incision was performed to exclude a peritoneal carcinomatosis. The laparotomy did not reveal any abdominal pathology except in the spleen. Neither peritoneal washing for cytologic examination nor peritoneal biopsies were taken. A total splenectomy was then performed (Figures 2 and 3). No intraoperative evidence of tumoral spreading in the abdominal cavity was observed, other than in the spleen. Our pathologic laboratory received a 550gram spleen, 9×11×17cm in dimensions, with fat tissue adhered to the spleen. Inside the spleen, we observed a tumor consistent with a high-grade metastatic endometrioid carcinoma grade 3 (Figures 4 and 5). Inside the adherent fat tissue, we observed another tumor with a diameter of 5cm, with the same characteristics as the splenic tumor.

The postoperative period was uneventful. Our patient was discharged from our ward a few days post surgery and was examined again two weeks later in our clinic, without any signs of operation-induced complications such as intra-abdominal bleeding, intra-abdominal infection or surgical site infection. CA-125 levels dropped to 33.3 unit/ml. The patient returned to the gyneco-oncologic clinic, and received another course of chemotherapy, again with paclitaxel (Taxol®) and carboplatin.

## Discussion

Splenic metastasis originates mostly from ovarian, breast, and lung carcinoma, and from melanoma. Solitary parenchymal splenic metastases are rare and often asymptomatic. Splenic metastasis from ovarian cancer is usually diagnosed during follow-up examinations, and is mainly based on elevation of CA-125 and imaging studies [7]. Mostly, ovarian carcinoma metastasizes through the peritoneal cavity to cause a visceral spreading that may include the capsule of the spleen. Solitary parenchymal metastases of the spleen are most probably generated through the hematogenic route, which is rare due to several mechanisms, including rhythmic contractions of the splenic capsule, the sharp angle of the splenic artery branching from the celiac artery, lack of afferent lymphatic vessels to the spleen and mostly - the inhibitory effect of the splenic microenvironment on the growth of metastatic cells [6].

In our case, we found no signs of peritoneal cavity metastases and the only metastases found were inside the spleen and in the adherent fat tissue, therefore, we assume that in this case the carcinoma metastasized hematogenically, and the tumor in the adherent fat tissue represents an extension of the splenic metastasis.

Increased diagnosis of solitary splenic metastasis is apparently due to improved and routine use of medical imaging [6]. In the current case, a rise in CA-125 prompted CT scanning. The six-year period that transpired between the original surgery to remove an ovarian carcinoma and detection of splenic metastasis is similar to the 57-month mean (range 28 to 88) between surgery for stage III disease and splenectomy reported in a series of six women with solitary splenic metastasis of ovarian carcinoma [8]. In a separate report, a solitary splenic metastasis of 12×8×5.5cm was detected by abdominal ultrasonography 20 years after surgery for an ovarian carcinoma. That patient was asymptomatic and tumor markers, including CA-125, were in the normal range [9].

Most cases of solitary splenic metastasis documented in the literature were diagnosed with stage III disease (FIGO classification), and underwent optimal tumor debulking [10]. The histological finding of the case described here was high-grade carcinoma consistent with metastatic endometrioid-type ovarian carcinoma, grade 3, stage 1A (FIGO classification). In Compérat *et al.*’s review, 17 of the 18 cases of splenic metastases with origins in ovarian cancer were serous cystadenocarcinoma and the remaining case was mucinous cystadenocarcinoma [6]. Elsewhere, a metachronous solitary splenic metastasis from an ovarian carcinosarcoma of dimensions 30cm×27cm×20cm was detected seven years after a bilateral salpingo-oophorectomy [11].

Most of the documented splenectomies were performed in an open versus laparoscopic procedure. Even though laparoscopic procedures have advantages such as faster recovery, shorter hospital stay and the possibility of earlier initiation of postoperative chemotherapy, debate persists as to the optimal treatment, mainly due to the possibility of peritoneal dissemination.

## Conclusions

The current case highlights that splenic metastasis should be suspected when an elevated CA-125 level is measured during follow-up of ovarian carcinoma, and should be confirmed using imaging studies such as ultrasound and CT scan. Splenectomy is the treatment of choice. Further studies are needed to determine the effectiveness of the laparoscopic versus open procedure [12,13].

## Consent

Written informed consent was obtained from the patient for publication of this case report and any accompanying images. A copy of the written consent is available for review by the Editor-in-Chief of this journal.

## Abbreviations

CA: cancer antigen; CT: computed tomography; FIGO: International Federation of Gynecology and Obstetrics.

## Competing interests

The authors declare that they have no competing interests.

## Authors’ contributions

DK (Dror Karni), a third-year resident, participated in the operation, conducted the literature review and wrote the first draft of this case report. DK (Doron Kopelman), the Head of General Surgery B’ at Haemek Medical Center, helped in writing the manuscript, and approved the final version. OAH was the leading surgeon who performed the operation, and a major contributor to writing the manuscript. All authors read and approved the final manuscript.
